# COVID Booster Shots: The Need of the Hour

**DOI:** 10.7759/cureus.19874

**Published:** 2021-11-24

**Authors:** Mani Maheshwari, Hemanthkumar Athiraman

**Affiliations:** 1 Hospital Medicine, Banner Health, Mesa, USA

**Keywords:** public health and safety, prevention, vaccine, covid-19, booster

## Abstract

Breakthrough cases of COVID-19 infections in fully vaccinated patients are rising. The number of patients requiring supplemental oxygen and airway management, along with multiple therapies, including remdesivir, baricitinib, steroids, and antibiotics, is more than ever among the vaccinated group. Despite being fully vaccinated for SARS-CoV-2 earlier this year, some are getting hospitalized for COVID-19 possibly due to waning immunity. Few such patients decline for the worse requiring intubation as they have not received the SARS-CoV-2 booster dose yet. This case report showcases four fully vaccinated patients who get hospitalized for COVID-19.

## Introduction

The rate of COVID-19-associated hospitalizations in fully vaccinated patients in all age groups is trending up [1]. Studies show that the effectiveness of Pfizer-BioNTech and Moderna vaccines in preventing SARS-CoV-2 infections significantly declines over time [2,3]. Though this study did not study the waning of vaccine efficacy, further research on this possibility is warranted as both mRNA vaccines were very effective against COVID early after vaccine administration, and effectiveness reduced significantly with time. It is essential to note an early study from Israel that did study the rate of infection in patients who previously received two doses of the Pfizer-BioNTech vaccine for SARS-CoV-2: immunity waned in all age groups [4]. This case report showcases fully vaccinated patients who were hospitalized for severe COVID-19.

## Case presentation

Case 1

**Figure 1:**
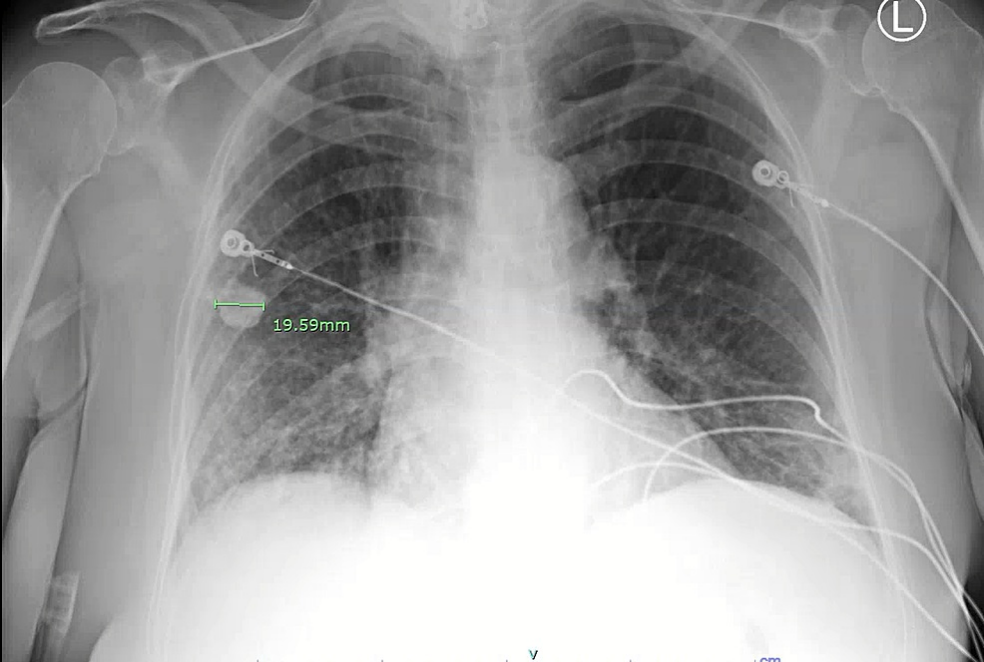
Chest X-ray on Admission

**Figure 2:**
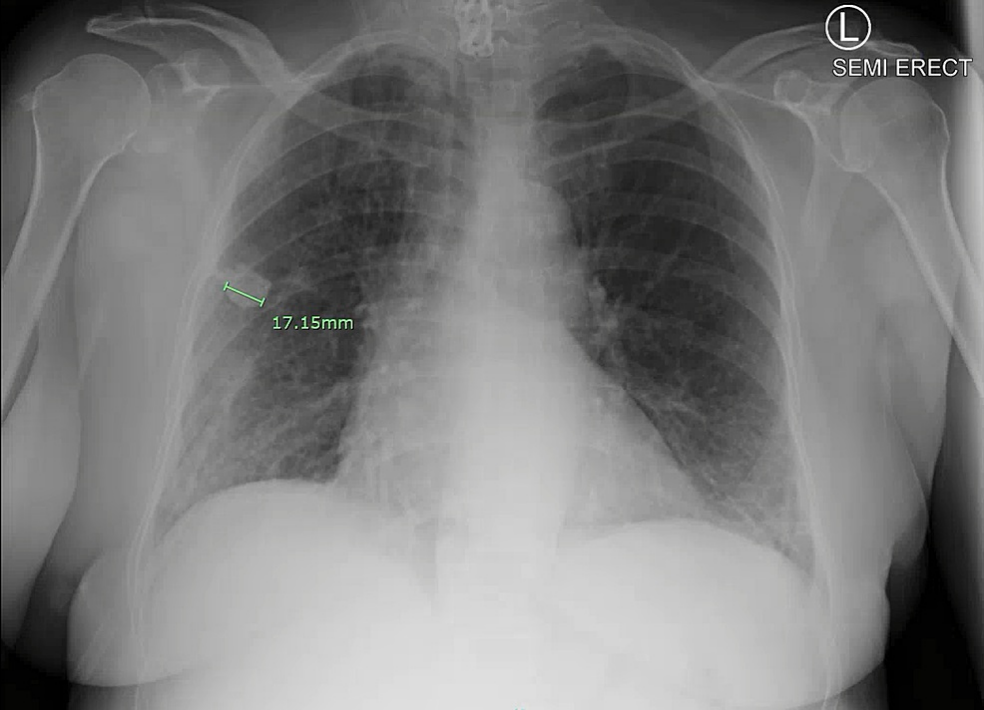
Repeat Chest X-ray 2 Days After Treatment

A 67-year-old female with a past medical history of chronic obstructive pulmonary disease and history of long-term tobacco abuse, who recently quit smoking, presented with shortness of breath, cough, myalgias, and malaise for one week. The patient had received two doses of Pfizer COVID vaccine, with the second dose in February 2021. In the ER, her vital signs were blood pressure (BP) 120/71, heart rate (HR) 78 bpm, respiratory rate (RR) 20 breaths/min, oxygen saturation 85% on room air, and afebrile. Laboratory assessment on admission is in Table 1. Nasopharyngeal swab for SARS-CoV-2 was positive. Chest X-ray on admission shows mildly patchy bibasilar pulmonary infiltrates and a calcified pulmonary nodule in the mid-right lung (2.0 cm) (Figure 1). The patient was admitted to the general medical ward and started on 6 L per minute of supplemental oxygen via nasal cannula, remdesivir, dexamethasone, furosemide, azithromycin, and enoxaparin for venous thromboembolism prophylaxis. Despite multiple measures, the patient did not improve, requiring more aggressive management. Repeat chest X-ray showed slight interval improvement of bilateral pulmonary infiltrates and needed 4-5 L per minute via nasal cannula (Figure 2). 

**Table 1:** Lab Work on Admission (+): Positive; (⬆): Higher than normal; (-): Not checked; AST: Aspartate transaminase; ALT: Alanine aminotransferase; NT-proBNP: N-terminal prohormone of brain natriuretic peptide

	Nasopharyngeal Swab for SARS-CoV-2 RNA	AST	ALT	Blood Glucose	NT-proBNP	D-dimer	C Reactive Protein
Case 1	+	⬆	⬆	⬆	⬆	Normal	⬆
Case 2	+	Normal	Normal	⬆	⬆	⬆	⬆
Case 3	+	Normal	Normal	⬆	-	⬆	⬆
Case 4	+	Normal	Normal	⬆	-	⬆	⬆

Case 2

**Figure 3:**
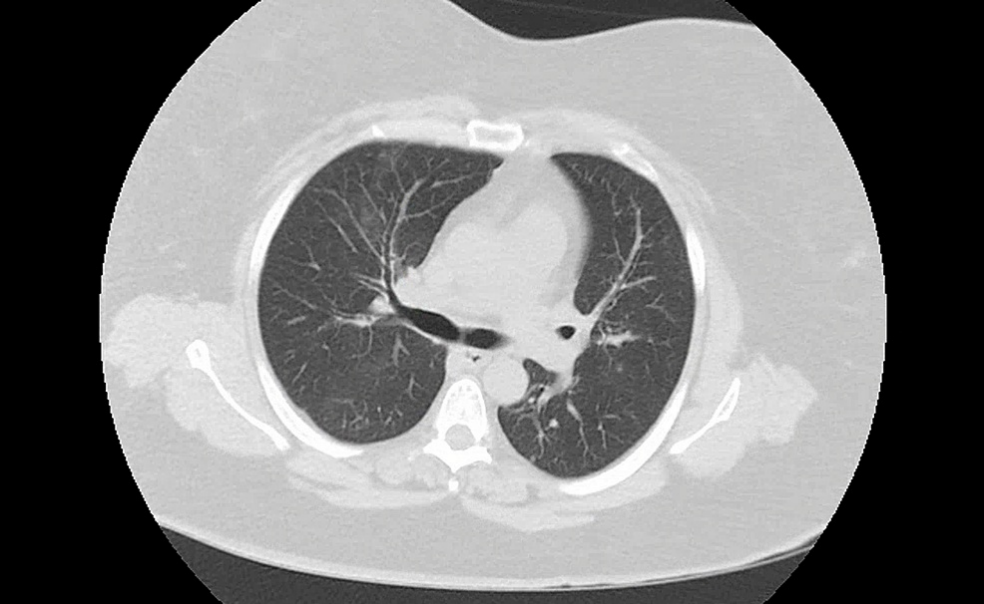
CT Chest on Admission

**Figure 4:**
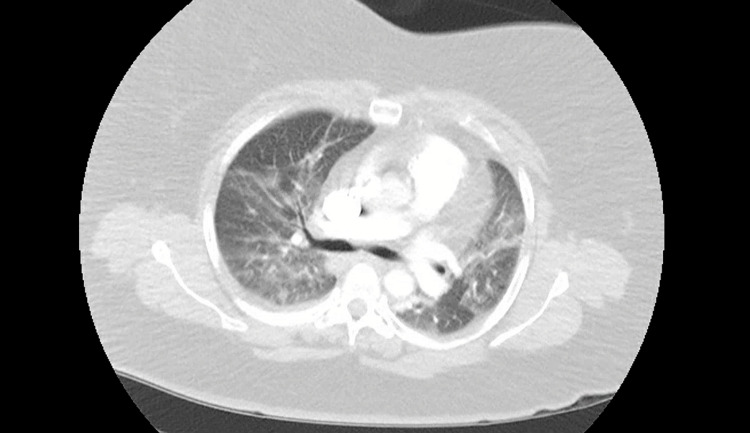
CT Chest 2 Days After Admission

A 58-year-old female with no significant past medical history presented with shortness of breath, fever, and cough for three days. The patient received two doses of the COVID vaccine, with the second dose in May 2021. In the ER, her vital signs were BP 105/96, HR 131 bpm, RR 20 breaths/min, oxygen saturation of 96% on room air, and febrile with a temperature of 102.0°F. Laboratory assessment is in Table 1. Nasopharyngeal swab for SARS-CoV-2 was positive. CT chest on admission shows no acute infiltrate and nonspecific nodules (Figure 3). The patient was admitted to the general medical ward and started on antibiotics, dexamethasone, and remdesivir. The patient developed worsening hypoxia on Day 2, and CT chest showed widespread airspace disease throughout the lungs (Figure 4). The patient required 4-5 L per minute via nasal cannula.

Case 3

**Figure 5:**
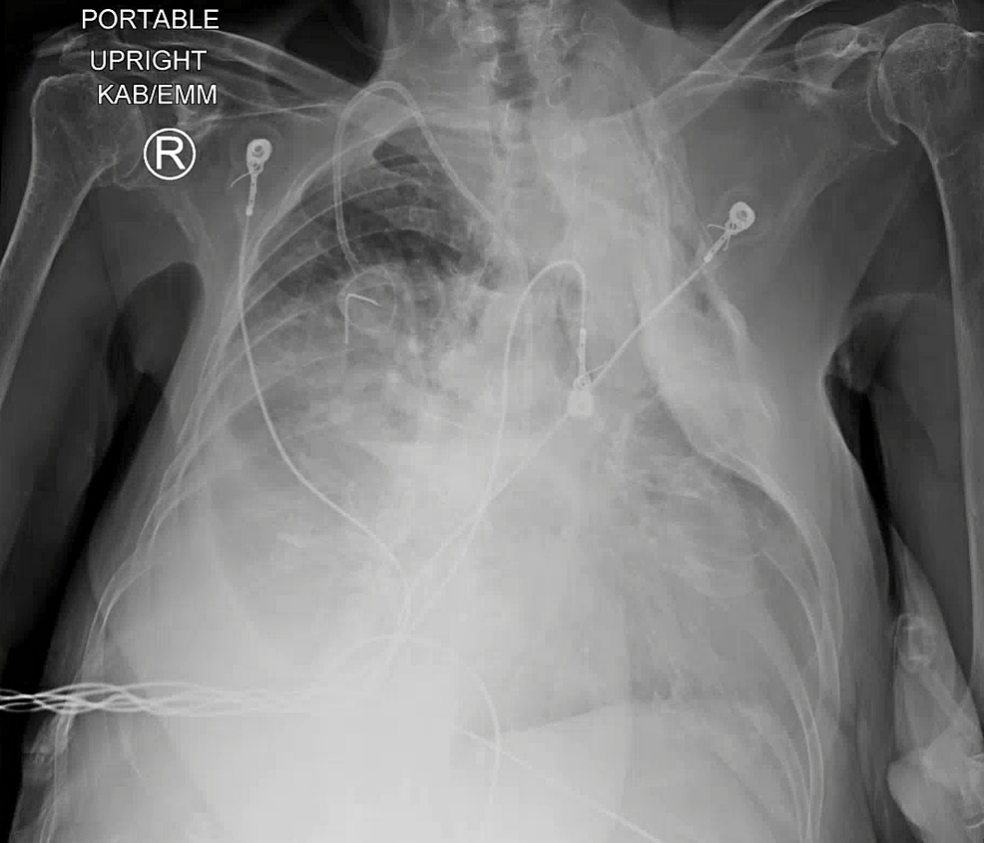
Chest X-Ray on Admission

**Figure 6:**
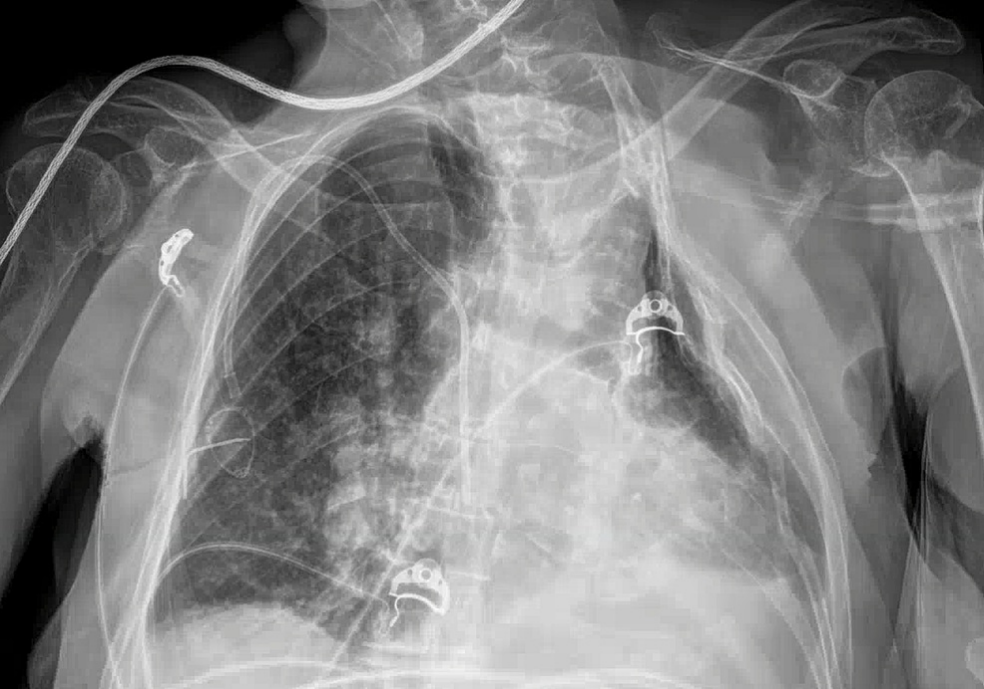
Chest X-Ray After Thoracentesis

An 84-year-old female with a past medical history of hypertension presented with weakness, dry cough, and shortness of breath for four days. The patient had received two doses of the COVID vaccine, with the second dose in March 2021. In the ER, her vital signs were BP 133/93, HR 103 bpm, RR 22 breaths/min, oxygen saturation of 96% on 40 L per minute of supplemental oxygen via high-flow nasal cannula, and afebrile. Laboratory assessment is in Table 1. Nasopharyngeal swab for SARS-CoV-2 RNA was positive. Chest X-ray on admission shows worsening right pleural effusion with new opacity obscuring the lower two-third of the right lung and a new pleural-based opacity in the left upper lobe (Figure 5). CT chest with contrast shows large right pleural effusion and associated right basilar consolidation and abdominal ascites. The patient was admitted to the telemetry unit and started on methylprednisolone, piperacillin-tazobactam, remdesivir, and baricitinib. The patient clinically deteriorated on Day 2 and was transferred to the intensive care unit for thoracentesis and possible intubation. Thoracentesis removed 1.95 L of bloody, serosanguineous fluid obtained, with partial resolution of the effusion (Figure 6). On Day 3, the patient developed septic shock, florid renal failure, and lethargy and was started on intravenous fluids and norepinephrine drip. Chest X-ray showed near-complete opacification of bilateral lung fields and subsequently was intubated. On Day 4, tense ascites were noted and the patient underwent paracentesis, which removed 4.25 L of bloody, serosanguinous fluid. Renal replacement therapy started. The patient was deemed to have a guarded prognosis with multiorgan failure.

Case 4

**Figure 7:**
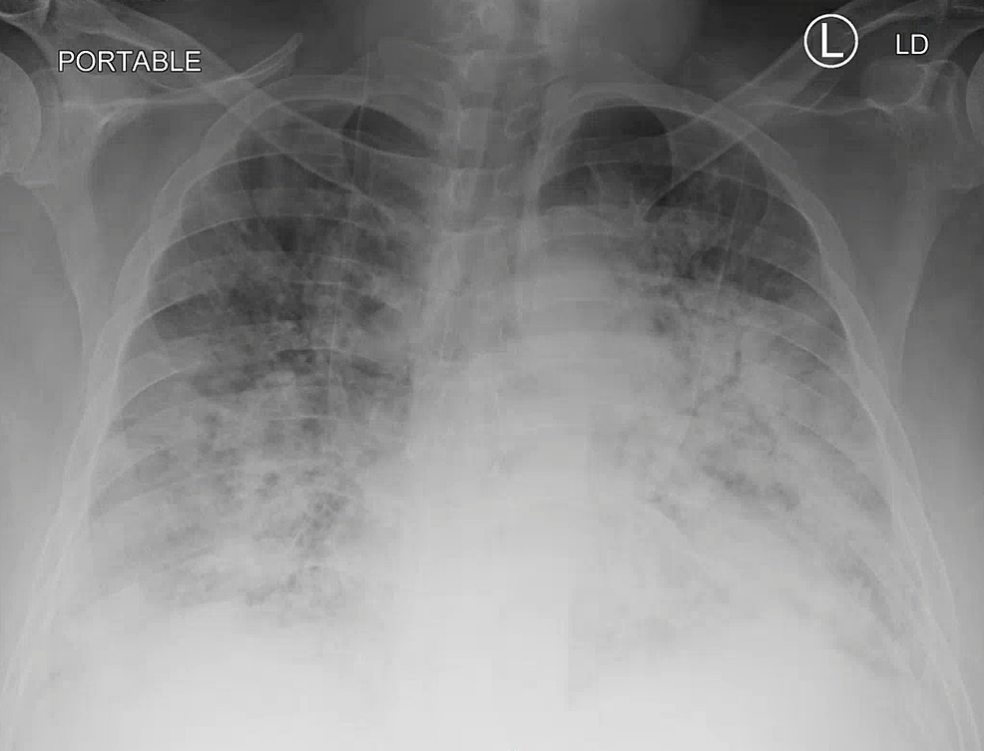
Chest X-Ray 1 Day Prior to Intubation

A 48-year-old male with a past medical history of type 2 diabetes mellitus and end-stage renal disease on hemodialysis presented with shortness of breath for three days. The patient was transferred from an outside facility where he was found to be hypoxic, saturating 79% on room air, chest X-ray showing infiltrates, and a positive nasopharyngeal swab for SARS-CoV-2 RNA. The patient had received two doses of the COVID vaccine, with the second dose in March 2021. Upon arrival to the general medical ward, the patient's vital signs were BP 132/79, HR 84 bpm, RR 18 breaths/min, oxygen saturation of 100% on 2-4 L per minute of supplemental oxygen via nasal cannula, and afebrile. Laboratory assessment is in Table 1. The patient was quickly weaned to room air, with SpO_2_ of 94-98%; hence, only supportive care was provided for COVID-19. On Day 1, the patient was found to have a right foot wound infection and was started on intravenous vancomycin and piperacillin-tazobactam. On Day 2, the patient was found to be hypoxic, put on 5 L per minute of supplemental oxygen via nasal cannula, and started on dexamethasone and remdesivir. Because of end-stage renal disease, the patient did not qualify for baricitinib. On Day 4, the patient required 10 L per minute of supplemental oxygen via high-flow nasal cannula, which he needed until Day 12, when we could start weaning down the supplemental oxygen over the next 3-4 days until Day 15 to room air. On Day 17, the patient was put back on 2 L per minute of supplemental oxygen via a nasal cannula which quickly escalated to 15 L on a nonrebreather mask within 2-3 hours, requiring the patient to get transferred to the intensive care unit on Day 18. At this time, he was put on bilevel positive airway pressure (BiPAP) with 40 L per minute of supplemental oxygen. Chest X-ray showed extensive diffuse airspace infiltrates, which significantly worsened from one week prior (Figure 7). He was dialyzed without improvement from a respiratory standpoint and then intubated on Day 19. Subsequently, the patient was restarted on dexamethasone and broad-spectrum antibiotics despite completing 14 days' worth previously.

## Discussion

The growing number of COVID cases among the vaccinated group is concerning [1]. The patients presented in this report were hospitalized for severe COVID-19 despite being fully vaccinated for SARS-CoV-2 RNA. Reasons could be multifaceted, from ever-changing/mutating virus, the waning of vaccine efficacy, and daily medications for other co-morbid conditions, including autoimmune diseases. Multiple studies in USA and Israel have shown that vaccine efficacy decreases over time [3-6]. Breakthrough COVID infections match the timing of about six months after the first group of high-risk workers and the elderly were vaccinated against SARS-CoV-2. This was the first time waning immunity was seen among the vaccinated population [6]. Further study is warranted to detect if the rise in waning immunity correlates with a rising number of breakthrough infections and if giving booster shots will decrease the number of breakthrough infections in the vaccinated population. 

With the winter and flu season quickly approaching, where indoor time increases, decreased masking, and waning immunity for those vaccinated for SARS-CoV-2, the Institute of Health Metrics and Evaluation projects a surge of COVID-19 infections this winter with over additional one million deaths [7]. If another COVID surge occurs, it will strain healthcare systems, facility resources, and staffing, which are already on the verge of breaking. The purpose of this case report is to present fully vaccinated patients who get hospitalized for severe COVID-19, highlighting the possibility of waning immunity resulting in breakthrough COVID-19 cases and discuss the potential need for booster shots to decrease such cases. 

## Conclusions

The need of this hour could be getting the booster shot against SARS-CoV-2 to potentially avoid another COVID surge this winter, given the risk of waning immunity discussed in the literature, if it has been six months or more from the date of the second vaccination.

## References

[1] (2021). CDC. COVID Data Tracker. https://covid.cdc.gov/covid-data-tracker.

[2] Nanduri S, Pilishvili T, Derado G (2021). Effectiveness of Pfizer-BioNTech and Moderna vaccines in preventing SARS-CoV-2 infection among nursing home residents before and during widespread circulation of the SARS-CoV-2 B.1.617.2 (Delta) variant - National Healthcare Safety Network, March 1-August 1, 2021. MMWR Morb Mortal Wkly Rep.

[3] Rosenberg ES, Holtgrave DR, Dorabawila V (2021). New COVID-19 cases and hospitalizations among adults, by vaccination status — New York, May 3-July 25. MMWR Morb Mortal Wkly Rep.

[4] Goldberg Y, Mandel M, Bar-On YM (2021). Waning immunity after the BNT162b2 vaccine in Israel. N Engl J Med.

[5] Thomas SJ, Moreira ED, Kitchin N (2021). Six month safety and efficacy of the BNT162b2 mRNA COVID-19 vaccine. medRxiv.

[6] Tartof SY, Slezak JM, Fischer H (2021). Effectiveness of mRNA BNT162b2 COVID-19 vaccine up to 6 months in a large integrated health system in the USA: a retrospective cohort study. Lancet.

[7] (2021). IHME: COVID-19 Projections. https://covid19.healthdata.org/global.

